# Exploring the Perception of Dental Undergraduate Students and Faculty on Environmental Sustainability in Dentistry: A Cross-Sectional Survey in 26 Dental Schools in Saudi Arabia

**DOI:** 10.3390/dj11040103

**Published:** 2023-04-12

**Authors:** Hasan Jamal, Abdullah A. Marghalani, Ahmed Al-Sharif, Albatool Shinawi, Balgis Gaffar, Ebtsam Abdullah Al-Edaili, Ghaliah Al-Baqami, Mayson AlQarni

**Affiliations:** 1 Independent Researcher, FDI Sustainability in Dentistry Task Team Member, Makkah 24371, Saudi Arabia; 2 Preventive Dentistry Department, College of Dental Medicine, Umm Al-Qura University, Makkah 24382, Saudi Arabia; aamarghalani@uqu.edu.sa; 3 Prosperity Clinics, Jeddah 23431, Saudi Arabia; a-20-aa@hotmail.com; 4 King Fahad Armed Forces Hospital, Jeddah 23311, Saudi Arabia; ashinawi@kfafh.med.sa; 5 Department of Preventive Dental Sciences, College of Dentistry, Imam Abdulrahman bin Faisal University, Dammam 34212, Saudi Arabia; Bgosman@iau.edu.sa; 6 Hail Health Cluster, Maternity Hospital, Hail 55471, Saudi Arabia; dr.e.aledaili@gmail.com; 7 College of Dentistry, Princess Nourah bint Abdulrahman University, Riyadh 11564, Saudi Arabia; ghalbaqami@pnu.edu.sa; 8 Department of Periodontology, King Saud University, Riyadh 11362, Saudi Arabia; maisonabdullah96@gmail.com

**Keywords:** sustainable dentistry, environment, sustainable development goals, education

## Abstract

There are few published studies assessing dental students’ and faculty’s perception regarding environmental sustainability in dentistry (ESD) and its existence within the dental curricula along with barriers and enablers for its integration. As far as the authors know, no published study has looked into this in Saudi Arabia, and this is what the current study aims to explore. A cross-sectional survey using validated online questionnaires for dental students and faculty was carried out in 26 dental schools in Saudi Arabia. The validated questionnaire utilized 25 questions using Qualtrics (XM) software. Both descriptive statistics and thematic analysis were included in the data analysis. ESD content is not formally embedded within the dental curricula in all 26 included universities in Saudi Arabia. Although the majority of students and faculty members agreed or strongly agreed on the importance of ESD as a whole and the importance of teaching it, more than 82% and 81% of students and faculty, respectively, demonstrated a lack of basic knowledge concerning ESD. The preliminary findings of the current study suggest that there is a high demand for incorporating ESD content in dental education in Saudi Arabia. To achieve this, resources and policy changes are necessary. A top-down approach is needed, including incorporating ESD into dental education standards, using evidence-based practices in revising infection control regulations, and providing institutional support through training, materials, and incentives. National dental associations and governments should provide practical solutions and actively support dental professionals in implementing ESD.

## 1. Introduction

Climate change and environmental pollution are considered the greatest health threats affecting the planet, humanity, and biodiversity [1]. Healthcare sectors negatively impact the environment through various aspects such as travel, waste, energy consumption, and procurements, with the oral health sector being a significant contributor [2,3,4]. In the United Kingdom (UK), the healthcare sector accounts for over 5% of the total greenhouse gas emissions produced in the country, with an estimate of more than 20 million tons per year [5]. In the United States (US), the healthcare sector produces 10% of the total greenhouse gas emissions [6]. Although the focus on oral healthcare delivery has always been aimed toward providing optimal care to patients, the environmental impact of the profession has been marginalised. The dental profession significantly contributes to the healthcare sector’s carbon footprint [7]. The three main greenhouse-gas-emission-contributing factors in dentistry are patient and staff travel (64.5%), procurement (19%), and energy (15.3%) [7]. Changing how dentistry is delivered has the potential to significantly contribute to the reduction of the environmental footprint, improve the quality of life of individuals and the quality of healthcare delivered, and help reach a circular economy throughout the entire healthcare sector [8].

The United Nations Agenda for Sustainable Development 2030 recognised that there is “an urgent call for action through all healthcare sectors” [9]. This was followed by ambitious goals and plans set by numerous countries around the globe, for example, those that followed the Paris Agreement in 2016 [10]. In April 2016, the Saudi Vision 2030 was first announced by Crown Prince Mohammed Bin Salman [11]. In 2021, the Kingdom of Saudi Arabia (KSA) pledged to reach net zero by the year 2060. Various environmentally sustainable initiatives in healthcare have been actioned in the KSA, such as the National Healthcare Sector Digital Transformation [12], with leading actions such as the establishment of the first virtual hospital (SEHA Virtual Hospital) [13].

Collectively, there is a call for dentistry as a profession to incorporate and integrate sustainable development goals in pursuit of healthy lives and well-being for all. Sustainability in oral healthcare is defined as “the provision of equitable, ethical, high-quality, inclusive and safe care, with appropriate, effective and efficient use of resources. Through this, the healthcare opportunities of current and future generations are respected and protected by actively minimizing negative environmental impacts” [14]. Therefore, to practice sustainable healthcare, healthcare professionals must become familiar with environmentally sustainable dentistry (ESD) concepts early on during their education and training. During the Association for Dental Education in Europe (ADEE) workshop in Berlin in 2019, a lack of teaching materials for instructors related to ESD was noted. Following the ADEE conference proceedings, there has been a growing interest in environmental sustainability in dental education [15].

Indeed, dental schools in the KSA have yet to develop ESD curricula. The Commission on Dental Accreditation (CODA) is yet to specify requirements for ESD in the dental training curricula. Although healthcare sustainability is of paramount importance within the Saudi Vision 2030, the authors are not aware of any ESD materials being taught in the dental school’s curricula in the kingdom. To our knowledge, no national study has been conducted in Saudi Arabia to explore current knowledge and drivers among dental students and faculty regarding ESD. Thus, this study aims to determine the presence of ESD in the dental curricula of all dental schools around the kingdom, explore the perception of dental students and faculty members regarding ESD, and identify possible barriers and enablers to its integration.

## 2. Materials and Methods

A cross-sectional survey using validated online questionnaires for dental students and faculty members was carried out in 26 dental schools in Saudi Arabia. The questionnaire was adopted from a previous study, which was conducted at Queen Mary University London and Harvard School of Dental Medicine, surveying both dental undergraduate students and faculty members [16]. The validated questionnaire utilized 25 questions using Qualtrics (XM) software. The survey was divided into several distinct sections. The initial block consisted of an information sheet that provided participants with a brief overview of the topic. Following this, participants were presented with a consent section in which they agree to participate in the study with full knowledge of its purpose and nature. They were informed that participation was voluntary and that they could withdraw from the study at any time. The second block of questions focused on participants. Familiarity with the subject matter consisted of five questions. This was followed by another set of five questions aimed at gauging participants’ awareness of ESD content in their dental curricula. This was followed by a set of additional questions that probed their awareness of existing policies, protocols, or events related to various ESD measures within their school. The third block of questions explored participants’ interest in teaching and learning about ESD and consisted of three questions. Faculty members were then asked additional questions pertaining to the barriers and enablers of integrating ESD content into their teaching practices. The final block of questions was designed to gather socio-demographic data from participants, with an additional feedback question included.

A mix of five-point Likert scales and free text with “yes/no” response styles were used. To minimize bias in the response selection of Likert scales, the order of the response options was altered, which started with negative (e.g., strongly disagree) and ended with positive (e.g., strongly agree). Inclusion criteria were undergraduate dental students and faculty members belonging to one of the twenty-six universities who agreed to participate. Questionnaires were distributed from 7 April 2022 through 18 October 2022. Invitations to participate were sent by email to undergraduate dental students and faculty members in the dental departments of all the universities and colleges. Ethical approval was obtained from Biomedical Research Ethics Committee at Umm Al-Qura University (IRB HAPO-02-K-012). Consent was obtained from all participants, and individual responses were anonymous, and any personal information was kept confidential. Both descriptive statistics and thematic analysis were included in the data analysis. 

Only responses that were completed and submitted in their entirety were subjected to analysis. In cases where there were multiple applicable responses to a given question, the corresponding response number was omitted, as its inclusion would not accurately reflect the overall number of respondents.

The following attempts were utilized to enhance the response rate: the respondents were informed about the purpose of the research and how their feedback would be used. The respondents were informed about the duration to complete the survey. A progress bar was used. Reminders were used at different intervals. The survey was optimized for all devices—desktop PCs, laptops, tablets, and mobile phones. 

## 3. Results

Responses were received from all 26 dental schools and colleges in Saudi Arabia. The total number of included responses of participants who completed and submitted the survey was 579 (353 students and 226 faculty members). In total, 343 participants were excluded due to incomplete or missing information.

The socio-demographic backgrounds of the involved participants are presented in Table 1.

Concerning familiarity with ESD content, the majority of students were “unfamiliar” to “somewhat familiar”, while faculty members, on the other hand, were “unfamiliar” to “moderately familiar” with ESD content (Table 2). The vast majority of students and faculty members thought that the dental profession as a whole has a responsibility to become environmentally sustainable. Moreover, the overwhelming majority of both students (87.2%) and faculty members (83.3%) “agree” or “strongly agree” on the importance of ESD as a whole, along with the importance of teaching it (82.3% and 81%), respectively (Table 2).

**Figure 1:**
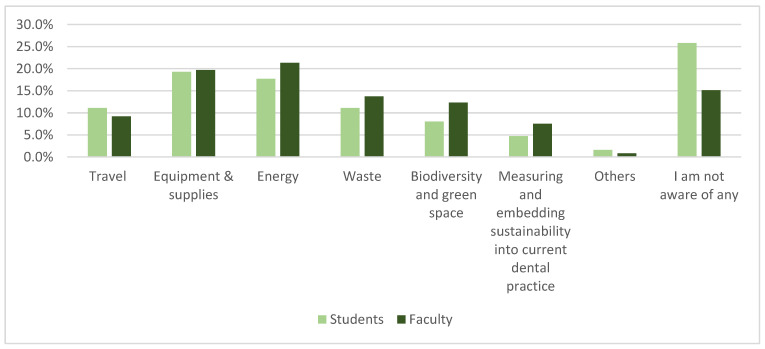
This bar chart demonstrates the awareness of existing policies, protocols, initiatives, or events concerning environmentally sustainable dentistry (ESD) by students and faculty members.

Regarding ESD policies, protocols, or initiatives, the majority of students and faculty members demonstrated a lack of awareness (Figure 1). Nonetheless, few students and faculty members shared comments on some policies and protocols embedded within their institutions, such as that on travel, equipment, energy, waste, and biodiversity. Regarding travel, few schools provide electric scooters and bicycles for students and faculty members to commute around the campus; few students, however, mentioned that it is provided in a limited number. Furthermore, others stated that their university encourages the utilisation of public transport. As for equipment, the main theme revolved around utilising reusable items, particularly stainless-steel impression trays. More comments on the energy aspect were mentioned by students and faculty members, such as turning off lights after lectures and clinical sessions (dental chair units) and using motion sensor light switches. As for waste management, a few comments stated that recycling bins are placed in all campus buildings; however, few stated that they are not used as intended. One student commented that they have a policy to recycle typodont teeth (artificial teeth) after using them. Additionally, others mentioned that computer-based exams are utilised instead of paper-based ones. Concerning biodiversity, a few students and faculty members stated that they do have green spaces as communal areas within the campus.

The overwhelming majority of students agreed on the relevance of ESD content for future dental practice and were interested in learning it (Table 3). Moreover, more than 81% of all faculty members expressed interest in introducing ESD content into the dental curriculum (Table 3).

Regarding the awareness of any included ESD contents in the dental undergraduate curricula, the majority of dental students and faculty members were not aware. Specifically, 73% of students and 83.6% of faculty members reported being not aware of any ESD contents, while 26.4% and 16.4% of students and faculty, respectively, were aware. Although few students and faculty members responded “yes” regarding being aware of ESD content included in their dental curriculum, the majority were not aware of a designated person or a department responsible for teaching it. Only a few students mentioned that dental public health departments are responsible for delivering indirect content concerning environmental sustainability; nonetheless, it is not integrated into the curriculum. As for the topics covered, the majority of students and faculty were not aware of any specific ESD topics covered in their curriculum.

**Figure 2:**
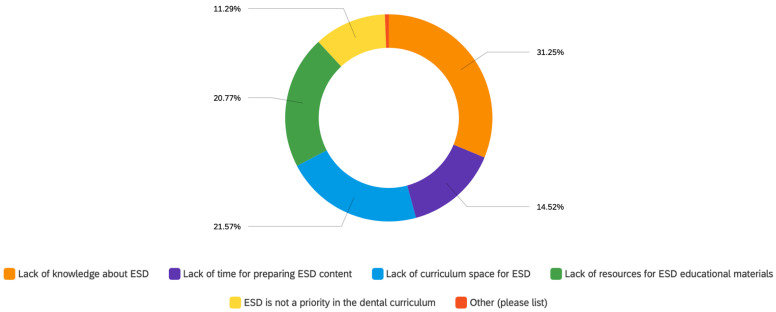
Faculty’s barriers to embedding environmentally sustainable dentistry (ESD) in the dental curricula.

The majority of faculty members thought that the main barrier to embedding ESD content into the curriculum was a lack of knowledge about ESD content, followed by a lack of curriculum space and a lack of educational material resources for ESD content (Figure 2). Few faculty members considered the lack of time in preparing ESD content as a barrier to embedding them. One faculty member stated that integrating a stand-alone subject into the curriculum will be difficult due to the existing policies and the required number of credits by the school.

**Figure 3:**
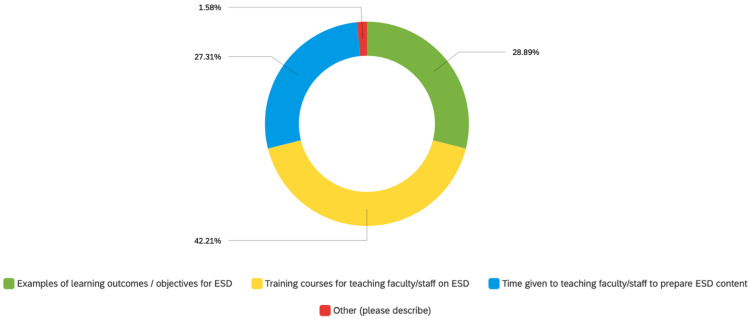
Faculty’s enablers to embedding environmentally sustainable dentistry (ESD) in the dental curricula.

The faculty members’ most noted enabler for embedding ESD content was training courses for teaching ESD content, followed by examples of learning outcomes and time given to teaching staff to prepare ESD content (Figure 3). Some faculty members stated that integrating ESD contents into existing modules such as within infection control will facilitate the integration process.

The majority of both students (51.3%) and faculty members (72.1%) emphasised the need to teach ESD in both classrooms and clinical settings. One faculty member suggested that experimental learning and “teach by example” would be an effective teaching opportunity for ESD content.

## 4. Discussion

ESD content is not formally embedded within the dental curricula in the 26 included universities and dental colleges in Saudi Arabia. Although the majority of students and faculty members agreed or strongly agreed on the importance of ESD as a whole and the importance of teaching it, more than 82% and 81% of students and faculty, respectively, demonstrated a lack of basic knowledge concerning ESD.

The findings were consistent with previously published studies in the literature that have explored this topic [16,17]. Gershberg et al. conducted a cross-sectional study including 378 dental students from 17 dental schools in the United States [17]. Despite the low response rate (5%), they found that although the majority of students thought that ESD is important, the majority of them were “slightly” to “not at all” knowledgeable about it. Meanwhile, similar results were demonstrated by another cross-sectional study assessing ESD content knowledge, enablers, and barriers in both UK and US dental curricula [16]. In this study, the questionnaires were directed towards dental students and educators at both Queen Mary University in London (QMUL) and Harvard School of Dental Medicine (HSDM). Similar to the current study, they found that ESD content was not formally integrated within the school’s curricula, and despite the interest of both students and educators for ESD content to be taught and embedded within the curricula, the majority demonstrated poor knowledge of the topic.

The barriers and enablers regarding ESD content as expressed by faculty members in the current study were also consistent with the previous work done by Joury et al. [16]. The identified enablers were the need for resources such as educational materials, training courses, and examples of learning outcomes and objectives. Barriers, on the other hand, were mainly focused on the lack of knowledge, curricula space, education materials, and time. A recent scoping review also identified the lack of knowledge as one of the main barriers as well as manufacturing challenges and the disposal of dental materials [18]. The same scoping review proposed the “linear economy supply chain” to implement sustainability in dental care from the manufacturing of materials, to dental care, and up through waste disposal [18]. Additionally, the review found that public awareness of the need to reduce pollution and decarbonize is at an all-time high; nonetheless, oral healthcare professionals’ awareness is much lower. On the other hand, it is crucial to address the observed knowledge gap in this study and previous ones. There is an ultimate need for the incorporation of sustainability in dental curricula as well as awareness campaigns targeting dental industries. Accreditation bodies should also consider sustainability in the curriculum and practice as a major requirement within dental schools.

Dental undergraduate students are mainly focused on the clinical aspect of the profession, and they lack the knowledge concerned with providing evidence-based information supporting and revolving around the environmental impact. This might be a consequence of reports such as that of the WHO-UNEP report on The Future Use of Materials for Dental Restoration [19]. The report highlighted that it is not a requirement for dental undergraduate students to be taught environmental and occupational health-related topics; subsequently, graduating dental professionals are not equipped with the proper knowledge to conduct, implement, or engage in research or practices related to environmental sustainability. A review in 2015 investigated the various initiatives taken by universities to increase awareness about sustainability [20]. The author reported that the first stage of awareness starts with organizational change. Alshuwaikhat et al. (2016) reported that universities within Saudi Arabia lacked sustainability-related projects within campuses as well as financial management practices [21]. Since then, the Kingdom of Saudi Arabia through a royal decree has launched multi-dimensional strategies related to sustainable development goals, one of which is the “Saudi Green Initiative” [22]. The initiative aims to target the overarching goal of the Saudi government to reach net zero by 2060 through various ambitious plans. Some of the highlights of the initiative are to plant 10 billion trees in the kingdom, reach 50% of Saudi electricity production from renewables by 2030, and to eliminate 130 million tons of carbon emission [22]. Achieving net zero is a global imperative for addressing the urgent threat of climate change, and numerous countries have committed to reaching this target. Thus, it is imperative for all stakeholders within the healthcare sector and especially the oral healthcare sector to also play their part. Collaboratively, there is a call for dentistry as a profession to incorporate and integrate sustainable development goals in pursuit of healthy lives and well-being for all.

Currently, there is a compounding acknowledgement across various sectors (stakeholders and supply chains) concerning the need for sustainable educational programmes. Universities, colleges, dental societies, and associations have both the responsibility and the opportunity to embed sustainable practices into their pedagogical curricula. Ongoing, rapidly evolving developments are being investigated to integrate sustainability within dental curricula in a broader context. Recently, the World Dental Federation (FDI) launched a Joint Stakeholder Statement for Consensus on Environmentally Sustainable Oral Healthcare. Through this, the consensus statement stressed the significance of involving all stakeholders in the interests of sustainability and advocated that dentistry as a profession should integrate sustainable development goals into daily practice and support a move to a circular economy [14]. For these goals to be achieved, ESD must be integrated formally into the curricula for oral health providers (OHPs). A range of key aspects regarding the inclusion of ESD in an undergraduate dental curriculum was addressed in this consensus statement. The integration of sustainability and prevention into paid-for healthcare systems was addressed as crucial to ensuring the health and well-being of both individuals and the planet. In addition to treatment, disease prevention should also be promoted as a continuous theme in education and professional development, including emphasizing the sustainable and effective use of materials and alternatives. Education for smart procurement, including bulk buying and making informed choices, was addressed as paramount to reducing waste. Policymakers should be educated on appropriate governance and legislation to support sustainable healthcare practices. Additionally, patient education should also include information on personal choices that may have a detrimental impact on the environment. Lastly, promoting sustainable travel was emphasized, and this included the use of public transportation and transportation that runs on clean energy, which ultimately can significantly reduce greenhouse gas emissions and support a healthier planet.

During the annual meeting of the Association for Dental Education in Europe (ADEE) in 2019, a special interest group (SIG) published a consensus view shedding light on how essential is ESD in dental education and that awareness should be raised concerning the need for educator’s support in developing ESD curricula [15]. Following the recent annual ADEE meeting of the SIG, in 2021, a consensus article was published [23], reporting the European consensus on suggested learning outcomes for ESD concerning the Graduating European Dentist curricula, providing recommendations for teaching ESD within existing OHP programmes, including methods of teaching and assessment. The outcomes of the ADEE SIG consensus meeting [23] suggested that the incorporation of ESD into OHP education poses challenges due to an already overloaded curriculum. To address this, a student-centred approach with practical resources and materials is necessary to help students and educators develop a basic understanding of ESD. Additionally, it was suggested that the integration of ESD should be longitudinal, both vertically and horizontally, across clinical and basic science stages across a given year. Furthermore, a consensus on various teaching methods was reached. These methods could include case-based learning, simulation-based learning, reflective writing, collaborative learning, and problem-based learning. It was emphasized that the curricula lead should regularly review content and practice for relevance while involving students in ESD incorporation processes.

It is widely recognized that dental professionals rely heavily on national associations and government regulations for guidance in their practice. These organizations provide standards and guidelines that aid in ensuring patient safety and promoting the highest level of care quality. However, with increasing awareness of environmental sustainability, and with the negative ramification that comes from the healthcare sector on the environment, these organizations must embed modules on sustainable oral healthcare within the dental curricula. This would equip dental professionals with the necessary knowledge and skills required to reduce the environmental impact of their practice, such as reducing waste, conserving water, and using eco-friendly materials. Further, it would align with the global shift towards sustainable practices and emphasize to the dental profession’s responsibility to protect the planet for future generations. Therefore, it is important for national dental associations and governments to provide oral healthcare professionals with achievable and practical solutions and to actively engage, evaluate, and support them.

There are some limitations in the current study. The current study targeted all dental schools in Saudi Arabia to obtain a representative sample to explore whether or not ESD is integrated into the dental curricula. However, it is worth mentioning that the survey results only reflect the opinions of those who responded to the survey and do not necessarily represent the views of all dental students and faculty members in Saudi Arabia. The response varied between the schools, and it might be that only individuals interested in ESD are those who participated in the survey. Nonetheless, the respondents’ low familiarity with ESD provides evidence against the likelihood of such a possibility. A further limitation of the current study was that the potential biases that might have emerged from “no-response” answers cannot be excluded, although attempts were made to reduce them. In addition, relating ESD awareness, opinion, or interest to a specific factor was beyond the scope of the current study.

## 5. Conclusions

Environmental sustainability in dentistry (ESD) is not currently included in the dental curricula at any of the 26 universities and colleges in the Kingdom of Saudi Arabia. However, the preliminary findings of the current study suggest that there is a clear desire among students and educators to incorporate ESD into dental curricula. Factors that would facilitate this include providing resources and implementing policy changes. A top-down approach including incorporating ESD into dental education standards, using evidence-based practices in revising infection control regulations, and institutional support through training, materials, and incentives is needed to effectively integrate ESD into dental curricula in Saudi Arabia. It is widely recognized that dental professionals rely on national associations and government regulations for guidance on changes in behaviour. Therefore, it is important for national dental associations and governments to provide oral healthcare professionals with achievable and practical solutions and to actively engage and support them.

## Figures and Tables

**Table 1:** Background profile of the participating students (N = 353) and faculty members (N = 226).

Variable	Students N (%)	Faculty N (%)
Gender		
Males	151 (42.8)	137 (60.2)
Females	193 (54.7)	84 (37.2)
Prefer not to answer	9 (2.6)	5 (2.2)
Age		
Less than 25 years old	283 (80.2)	7 (3.1)
Between 25–34 years old	67 (19)	66 (29.2)
Between 35–44 years old	2 (0.8)	98 (43.4)
Between 45–54 years old	1 (0.3)	40 (17.7)
Between 55–64 years old	-	12 (5.3)
More than 65 years of age	-	3 (1.3)
Study year		
1st year	24 (6.8)	-
2nd year	36 (10.2)	-
3rd year	40 (11.3)	-
4th year	62 (17.6)	-
5th year	65 (18.4)	-
6th year	126 (35.7)	-
Faculty members’ rank		
Demonstrators	-	13 (5.7)
Lectures	-	45 (20)
Assistant professors	-	112 (49.5)
Associate professors	-	29 (13)
Professors	-	15 (6.6)
Others	-	12 (5.3)

**Table 2:** Student and faculty familiarity with ESD and their opinions about the importance and professional responsibility for ESD.

Component	Overall	
	Students N (%)	Faculty N (%)
Familiarity with ESD		
Not at all	57 (25.2)	88 (24.9)
Slightly	38 (16.8)	64 (18.1)
Somewhat	57 (25.2)	80 (22.7)
Moderately	53 (23.5)	88 (24.9)
Extremely	21 (9.3)	33 (9.4)
Profession’s responsibility for ESD		
Strongly agree	115 (50.9)	122 (35.6)
Agree	73 (32.3)	161 (45.6)
Neither agree nor disagree	31 (13.7)	60 (17)
Disagree	4 (1.8)	7 (2)
Strongly disagree	3 (1.3)	3 (0.9)
ESD is important		
Strongly agree	144 (50.4)	136 (38.5)
Agree	83 (36.7)	158 (44.8)
Neither agree nor disagree	26 (11.5)	55 (15.6)
Disagree	1 (0.4)	3 (0.85)
Strongly disagree	2 (0.9)	1 (0.3)
ESD teaching is important		
Strongly agree	97 (42.9)	149 (42.2)
Agree	89 (39.4)	137 (38.8)
Neither agree nor disagree	28 (12.4)	59 (16.7)
Disagree	8 (3.5)	6 (1.7)
Strongly disagree	4 (1.8)	2 (0.6)

**Table 3:** Students’ and faculty’s interest in ESD teaching and learning.

Component	Overall	
	Students N (%)	Faculty N (%)
ESD relevance for future dental practice		
Strongly agree	117 (33.1)	-
Agree	145 (41.1)	-
Neither agree nor disagree	78 (22.1)	-
Disagree	8 (2.3)	-
Strongly disagree	5 (1.4)	-
Interested in learning ESD		
Strongly agree	141 (39.9)	-
Agree	137 (38.8)	-
Neither agree nor disagree	64 (18.1)	-
Disagree	8 (2.3)	-
Strongly disagree	3 (0.9)	-
Interested in introducing ESD into the dental curriculum		
Strongly agree	-	111 (49.1)
Agree	-	73 (32.3)
Neither agree nor disagree	-	31 (12.7)
Disagree	-	8 (3.5)
Strongly disagree	-	3 (1.3)

## Data Availability

Not applicable.
